# A Survey of Accidental Hypothermia Knowledge among Navy Members in China and the Implications for Training

**DOI:** 10.3390/ijerph13030315

**Published:** 2016-03-11

**Authors:** Shuang Li, Chen Qiu, Wenwen Shi, Yan Huang, Li Gui

**Affiliations:** Emergency Nursing Department, School of Nursing, 2nd Military Medical University, Shanghai 200433, China; shiny0820@foxmail.com (S.L.); qiuchenqiuying@163.com (C.Q.); xiaowenz@126.com (W.S.); yan8811@hotmail.com (Y.H.)

**Keywords:** accidental hypothermia, AH, Navy, health survey, China

## Abstract

*Objectives*: Accidental hypothermia (AH) is a potentially life-threatening condition that can lead to significant morbidity and life-long effects. Navy personnel are always at a greater risk of AH due to frequent outdoor work, wilderness exposure, prolonged immobility and exhaustion. The purpose of the survey was to assess Chinese Navy members’ awareness of AH and to make recommendations with regard to better measures for improving it. *Methods*: 111 Navy members completed a written questionnaire that was subsequently analyzed. *Results*: 30.6% of the respondents have experienced AH and 64.9% rated their knowledge of AH as “low” or “none”. Over half of them identified the initial symptom of AH as obvious shivering (69.4%) and apathy (45.0%). As for the aggravate symptoms, 60.9% chose the wrong answer of more obvious shivering instead of the right one—absence of shivering (5.4%). In the case of the treatment of mild AH, more than half of the respondents chose the wrong answers. *Conclusions*: This study suggests that the basic skills of recognition and treatment of AH are inadequate in the Chinese Navy. Further work is required to develop a systematical, comprehensive and corresponding education method that would promote correct actions during AH.

## 1. Introduction

Accidental hypothermia (AH) is defined as an unintentional drop in core body temperature to 35 °C or below, occurring in patients with normal thermoregulatory mechanism but become hypothermic due to extremely cold stress, which can be classified as mild (32 °C–35 °C), moderate (28 °C–32 °C), severe (20 °C–28 °C) and profound (<20 °C) [1,2]. Although infrequent, it is a preventative life-threatening condition which can contribute to significant morbidity and poor prognosis. Concerning the morbidity and mortality rates of AH, the data varies from country to country. In the United States, approximately 600 to 700 deaths are due to AH each year, and the annual death rate ranges from 0.2 to 0.5 per 100,000 populations [3,4,5,6]. In England and Wales, AH was written on the death certificates of 236 people in 2003 [7]. 

There are multifactorial causes of AH, such as exposing to cold environment (snow, wind, water, or altitude), immersing in cold water, immobilizing postures in cold scenarios, or suffering from low temperatures without adequate protection. Interestingly, AH can even occur in temperate climates [8]. It is reported that, in the USA, the number of AH cases in warm states is almost as great as that in cold states [9,10].

The maritime environment that can bring hazards to military operations or adventurous training has the great potential of exposing Navy personnel to AH. Navy personnel, especially those engaging in combat-specific occupations, are always at a greater risk for AH due to frequent outdoor work, wilderness exposure, prolonged immobility and exhaustion, and they usually lack access to plentiful medical resources. One published report showed that, in the USA, incidence of hypothermia of the Navy rose in 2013–2014 (17, rate: 5.3 per 100,000 person-years), which was the highest of the past five years [11]. It is, therefore, of great importance for Navy personnel to have a basic knowledge of AH. In reality, there are no published studies about AH in military personnel who have served in the Chinese Navy.

This investigation was intended to assess the Chinese Navy members’ knowledge of AH, which included symptomatology, prevention, treatment, and the resources used to gain this awareness. Through this assessment, we hope to find better methods of education to decrease the morbidity and mortality of AH. 

## 2. Materials and Methods

This study was a cross-sectional survey of Chinese Navy personnel which was carried out in January 2015. Ethical approval was obtained from the Second Military Medical University in China (CHEC2015-037).

### 2.1. Research Participants

Participants were recruited from a unit of the active-duty Navy members in the northeastern part of China by convenience sampling. Subjects who refused to take part or had a professional background in health care were excluded from the study. All of them provided written informed consent to participate the survey. They were then asked to fill out and return the questionnaire. The responses were anonymous and voluntary. 

### 2.2. Data Collection

The questionnaire (see Appendix) was self-designed mainly according to the Wilderness Medical Society Practice Guideline, Hypothermal Medicine [1]. Demographic information (see Table A1) included questions about age, gender, nationality, military-related information, education level, and medical background. The AH knowledge (see Table A2) that was assessed included origins of AH knowledge, the experience and emergency first response (EFR) for AH, the knowledge of symptoms, treatment as well as risk factors of AH. The questionnaire consisted of ten multiple-choice questions requiring either single or multiple answers and one open-ended question. The filled-out data collection forms were reviewed and categorized by the author.

### 2.3. Statistical Analysis

Statistical analysis was performed with SPSS Version 20.0 (IBM, Armonk, NY, USA). Continuous variables are reported as means ± standard deviation. Categorical variables are reported as counts and the percentages of people responding appropriately to the question compared to the total number of participants. To check for confounding factors, we conducted *t*-tests and analysis of variance tests. The chi-square test was used with varying degrees of freedom depending on the specific item. A *p* value of less than 0.05 was considered to be significant. 

## 3. Results

A total of 120 subjects were approached, and 111 were finally included in the study (92.5%), since 5 participants who had medical background and 4 participants who did not complete the questionnaire were excluded from the investigation. Demographics of the respondents are shown in Table 1.

In the case of the normal body temperature, the majority of Navy members (88.3%, *n* = 98) responded exactly by ticking the right choice of 37 °C, whereas fewer selected the wrong choice of 35 °C (10.8%, *n* = 12) and 39 °C (0.9%, *n* = 1). 

The participants were aware of their lack of knowledge, as 64.9% (*n* = 72) rated their knowledge of AH as “low” or “none”. A variety of resources were used by the respondents to gain information about AH, but the most frequently used resources were books/magazines/newspapers (28.8%, *n* = 32), family (20.7%, *n* = 23) and Internet (13.5%, *n* = 15). Military medical education (13.5%, *n* = 15) and television (18.9%, *n* = 21) were identified as the origin of hypothermia knowledge, whereas 20.7% (*n* = 23) did not respond.

In response to the AH experience, 30.6% (*n* = 34) have experienced AH. Regarding the EFR for AH, 12.6% (14/111) of the respondents thought they were able to assess the patient’s condition and make some interventions, whereas 82.9% (92/111) selected the choice of calling for help from a military surgeon or medical corpsmen. The rest (2.7%, 3/111) chose to do nothing to help the victim. 1.8% (*n* = 2) did not respond. 

In Table 2, the numbers and percentages of correct answers about symptoms of AH are presented. There were no significant differences in the proportion of responses within each question defining from demographic characteristic. 

The details regarding the numbers and percentages of correct answers about treatment of AH can be found in Table 3, in which the accuracy is not as satisfying as expected. The optimal food for AH was identified as candy by 54.1% of the respondents (60/111) that were technically correct, with 30.6% (34/111) preferring “beef jerky.” Meanwhile, 3.6% (*n* = 4) did not respond. Again, there were no significant differences in the proportion of responses within each item defining from demographic characteristic. 

As for the risk factors of hypothermia, the response rate of this question was very low (40.1%, 45/111), probably because it is an open-ended question. However, according to the existing answers, the respondents listed cold weather (21.6%, 24/111), prolonged training (19.8%, 22/111), outdoor work (18.9%, 21/111) and illness (5.4%, 6/111) as the risk factors of AH.

## 4. Discussion

To our knowledge, this is the first study to assess the knowledge of AH in the Navy of the People’s Liberation Army, China. There have been several previous studies to assess the knowledge in general public and medical personnel, revealing both that there is a lack of AH knowledge among them and that even hospitals have no protocol for treating AH [12,13]. Navy personnel are always under great risk of AH. Although uncommon, there were 30.6% of the respondents who had experienced AH. However, a high proportion (64.9%) rated their knowledge of AH as “low” or “none”, reflecting that accurate educational messages may need to be targeted to this group. 

### 4.1. Symptom Recognition

Undoubtedly, prompt recognition of AH is necessary for improving prognosis and reducing mortality. The initial symptoms of AH include shivering, apathy and ataxia. As the victim’s condition deteriorates, symptoms can progress to confusion, slower pulse and respiration, loss of fine motor skills, and the absence of shivering [14]. 

The findings of this study reveal that the information most Navy members mastered about symptoms of AH is not complete and accurate, clearly indicating the need to change Navy members’ cognition about AH symptomatology. 69.4% of the respondents could identify “obviously shivering”, whereas less than half of them (45.0%) were aware that “apathy” is also a classic symptom of AH. In the case of aggravated symptoms, many subjects (55.9%) elicited the right choice as “unconscious or coma”, while very few (5.4%) recognized “absence of shivering” as the correct option. Further education should be emphasized on these specific points, especially the incorrect answers with high proportion. For example, as hypothermia worsens, the shivering will stop instead of increase or become more noticeable. 

### 4.2. Treatment Knowledge and Rewarming Strategies

Advanced knowledge of AH treatment is a prerequisite to providing optimal care for AH patients. However, this study clearly shows that most Navy personnel misunderstand how AH is properly treated, probably resulting from incorrect information being consistently presented over many years and inadequate education. 

In the case of the optimal food for sufferers of AH, about 1/3 respondents (30.6%) regarded beef jerky as the preferred choice, presumably because they think it is full of energy and because men prefer meat to candy. In reality, however, sugar can provide calories to help fuel shivering persons more quickly. 

General treatment options for AH victims include removal from the cold environment, prevention of further heat loss and the commencement of rewarming [15,16]. Most importantly, the choice of the appropriate rewarming methods and its optimal timing are the key issues in the successful treatment of hypothermia. 

There are some critical warnings to be highlighted during rewarming. First, movement or significant warming of the extremities, as with warm-water immersion, increases blood flow to colder tissues, cooling blood that then returns to the core circulation. This can contribute to core temperature afterdrop and has the potential to cool the heart and to increase the risk of ventricular fibrillation (VF) [17]. Thus, rewarming of the trunk should be performed before that of extremities to minimize the risk of core temperature afterdrop. Besides this, external heat is most effective if concentrated on the axillae, chest, and back—those areas with the highest potential for conductive heat transfer. 

Second, as for a shivering patient, exercise should be delayed to protect against afterdrop and shivering should not be restrained. Then, once the patient is in a warm environment, clothes should be cut off rather than removed manually. Undoubtedly, shivering is an effective method of rewarming a patient who is cold, but not someone who has hypothermia, however severe [1]. Some studies have demonstrated that vigorous shivering can increase heat production by 5 to 6 times the resting metabolic rate and up to 50% of maximal metabolic rate [18,19]. Additionally, a warm shower or bath for rewarming, even if a patient appears to be only mildly hypothermic, should not be used, as it may increase afterdrop and potentially cause hypotension.

Furthermore, as hypothermia deteriorates, patients usually require more aggressive strategies, such as active internal warming and active extracorporeal rewarming, because external warming (e.g., immersion in warm water) is not effective and will prolong the return to normal temperature.

### 4.3. Risk Factors of AH

It is believed that AH is a preventable disease; thus, the knowledge of risk factors is of greatest importance. As mentioned before, hypothermia commonly results from impaired cognition (drug- or alcohol-induced), immobility, wilderness exposure with inadequate shelter and clothing, or prolonged exposure to low temperatures. Although the response rate is not high as we expected, the answers they listed are hardly acceptable. Nonetheless, accurate and complete educational messages may need to be targeted toward this focus.

### 4.4. Navy Education

This study, based on a small sample of Navy personnel, suggests that the basic skills of recognition and treatment of AH are inadequate in the at-risk population. Thus, more work should be required to develop a systematical, comprehensive and corresponding education method in Navy as soon as possible. 

There are several strategies for improving the distribution of information to the Navy. First, combine military education with various media resources. Additionally, the Navy could set an Accidental Hypothermia Unit or special treatment group to guide the wilderness response to hypothermia victims if possible. The education could be thus more niche targeting. Educational materials should highlight common misconceptions and blind spots, and distinguish effective rewarming methods from the harmful ones. Aside from this, it may be beneficial to equip the Navy with such devices as the Hypothermia Prevention Management Kit (HPMK) [1].

## 5. Conclusions

This is the first study to assess the knowledge of AH in the Navy of the People’s Liberation Army. Our study suggests that the basic skills of recognition and treatment of AH are inadequate in the at-risk population-Chinese Navy. Therefore, further work is required to develop a systematical, comprehensive and corresponding education method that would promote correct actions during AH.

## 6. Limitations

The major drawbacks in our study were stemming primarily from its small scale. The data would be more generalized if the sample size were larger and not restricted to one military area. In addition to sample size, we limited the survey to convenience methodology. 

## Figures and Tables

**Table 1:** Demographic characteristics of participants (*n* = 111, all male).

Characteristic	Number of Navy Personnel (% of Total)
Gender	
Male	111 (100)
Female	0 (0.0)
Age group	
≤20	21 (18.9)
21–30	73 (65.8)
31–40	16 (14.4)
No response	1 (0.9)
Nationality	
Han	110 (99.1)
Hui	1 (0.9)
Attended time	
≤5 years	55 (49.5)
6–10 years	30 (27.0)
11–15 years	18 (16.2)
16–20 years	6 (5.4)
No response	2 (1.8)
Degree of education	
Bachelor’s degree or above	15 (13.5)
College degree	46 (41.4)
Vocational degree	23 (20.7)
High school diploma	26 (23.4)
Junior high school diploma or below	1 (0.9)
Relatives work in medical or healthcare field	
Yes	44 (39.6)
No	67 (60.4)

**Table 2:** Choice of symptoms (*n* = 111).

Questions	Number of Navy Personnel	Percentage of Total (%)
The primary symptoms of hypothermia		
① Obvious shivering *	77	69.4
② Unresponsive	18	16.2
③ Hypalgesia	31	27.9
④ Apathy *	50	45.0
⑤ Others	2	1.8
No response	5	4.5
The correct answers (①, ④)	26	23.4
Which symptoms indicate the exacerbation of hypothermia		
① Shivering more obviously	68	61.3
② Slower pulse *	38	34.2
③ Loss of consciousness or coma *	62	55.9
④ Faster respiratory rates	25	22.5
⑤ Absence of shivering *	6	5.4
⑥ Others	2	1.8
No response	4	3.6
The correct answers (②, ③, ⑤)	1	0.9

Note: * represents the correct answer.

**Table 3:** Treatments of hypothermia (*n* = 111).

Questions	Number of Navy Personnel	Percentage of Total (%)
1. Encourage him to run for heat production when someone is shivering ceaselessly because of coldness.		
True	40	36.0
False *	70	63.1
No response	1	0.9
2. Be quick to rewarm the victim by means of putting hot packs on his extremities if you find someone is shivering and incapacitated because of coldness.		
True	87	78.4
False *	23	20.7
No response	1	0.9
3. Help him take warm baths when someone appears to be mildly hypothermic.		
True	33	29.7
False *	77	69.4
No response	1	0.9
4. Stop training and immerse him in warm water immersion when someone with hypothermia has decreased level of consciousness and weak pulse.		
True	89	80.2
False *	21	18.9
No response	1	0.9
5. Discourage the patient from shivering when he is mildly hypothermic during training in cold weather.		
True	45	40.5
False *	63	56.8
No response	3	2.7
6. Help him drink warm high-carbohydrate liquids when an alert patient who is shivering after exposing to a cold environment.		
True *	90	81.1
False	18	16.2
No response	3	2.7

Note: * represents the correct answer.

## Appendix Accidental Hypothermia Questionnaire

We are conducting a survey regarding your knowledge of accidental hypothermia. We ask if we could please take 10 min of your time to help fill out this survey. The information you provide is completely confidential. Wish you to make the truthful answers based on your own condition. Thank you for your participation.

**Table A1:** Demographic Information.

A.1. Gender: □ Male □ Female
A.2. Age:
A.3. Nationality:
A.4. How many years on duty?
A.4. How many years on duty?
A.5. What’s your unit?
A.6. What’s your military rank?
A.7. What’s your post of duty?
A.8. What’s the highest education level that you have obtained?
□ Bachelor’s degree or above □ College degree □ Vocational degree
□ High school diploma □ Junior high school diploma or below
A.9. Have you ever worked in medical or healthcare filed? □ Yes □ No
A.10. Are there any medical staff in your family, relatives or friends?
□ Yes □ No

**Table A2:** The knowledge of accidental hypothermia.

B.1. Which one is the normal body temperature? (Please check one)
□ 35 °C □ 37 °C □ 39 °C □ 41 °C
B.2. Have you or your companions ever experienced accidental hypothermia?
□ Yes □ No
B.3. How would you rate your knowledge of accidental hypothermia? (Please check one)
□ 5 –Expert □ 4 – High □ 3 – Medium □ 2 – Low □ 1 – None (If you choose 1, you can skip to question 5.)
B.4. What resources have you used to obtain the hypothermia knowledge?
□ Military medical education □ Television
□ Information from family and friends □ Books/ Magazines/ Newspapers
□ Internet □ Other:
B.5. Which is the primary symptom of hypothermia?
□ Obvious shivering □ Unresponsive □ Hypalgesia
□ Apathy □ Other
B.6. Which of the following indicates thee probability of progressing to a more severe hypothermia state?
□ Shivering more obviously □ Slower pulse
□ Loss of consciousness or coma □ Faster respiratory rates
□ Absence of shivering □ Other:
B.7. What would you do if someone is in a state of coma due to hypothermia?
□ Too nervous to do anything
□ Call for help from military surgeon or medical corpsmen
□ Assess the patient’s condition and take some interventions
B.8. Which is the best food he should eat when someone with hypothermia is listless and shivering?
□ Compressed biscuit □ Candy □ Bread
□ Beef jerky □Other:
B.9.1. Encourage him to run for heat production when someone is shivering ceaselessly because of coldness.
□ True □ False
B.9.2. Be quick to rewarm the victim by putting hot packs on his extremities if you find someone is shivering and incapacitated because of coldness.
□ True □ False
B.9.3. Help him take warm baths when someone appears to be mildly hypothermic.
□ True □ False
B.9.4. Stop training and immerse him in warm water when someone with hypothermia has decreased level of consciousness and weak pulse.
□ True □ False
B.9.5. Discourage the patient from shivering when he is mildly hypothermic during training in cold weather.
□ True □ False
B.9.6. Help him drink warm high-carbohydrate liquids when an alert patient is shivering after exposing to a cold environment.
□ True □ False
B.10. In what situations you need to be alert for hypothermia when the troops are conducting training or military operations? Please list as many as possible, such as long time diving in cold water.
